# A Study of the Possibility of Producing Annealed and Metallized Pellets from Titanomagnetite Concentrate

**DOI:** 10.3390/ma17215338

**Published:** 2024-10-31

**Authors:** Andrey N. Dmitriev, Galina Y. Vitkina, Victor G. Zlobin, Elena A. Vyaznikova, Larisa A. Marshuk, Yulia E. Burova, Roman V. Alektorov, Vladimir V. Kataev

**Affiliations:** Laboratory of Pyrometallurgy of Reduction Processes, Institute of Metallurgy of the Ural Branch of the Russian Academy of Sciences, 101 Amundsen St., 620016 Ekaterinburg, Russia; andrey.dmitriev@mail.ru (A.N.D.); vjaznikova@mail.ru (E.A.V.); ferro@ural.ru (L.A.M.); menestrelfox@gmail.com (Y.E.B.); alektorov-rv@mail.ru (R.V.A.); kataev.5959@mail.ru (V.V.K.)

**Keywords:** processes of titanomagnetite beneficiation, technology, iron ore concentrate production, pellet creation, blast furnace operation, metallization, phase composition, iron ore concentrate properties

## Abstract

The current intensive development of steelmaking is being impeded by a scarcity of pure scrap. The potential to replace pure scrap with metallized raw materials that are naturally alloyed with vanadium and titanium, such as annealed unfluxed titanomagnetite pellets, could facilitate the achievement of key objectives in metallurgical development, particularly in the smelting of electric steel. The objective of this research was to produce annealed and metallized pellets from titanomagnetite concentrate under laboratory conditions, with the intention of further processing them as a commercial product in a blast furnace or as an intermediate product for the production of hot briquetted iron (HBI). The results demonstrate that pellets derived from titanomagnetite concentrate exhibit sufficient compressive strength (up to 300 kg/pellet) and a degree of metallization exceeding 90%, which aligns with the requirements for electric steelmaking. The suitability of pellets derived from titanomagnetite concentrate for use in both blast furnaces and metallization processes has been corroborated.

## 1. Introduction

The principal means of producing steel is through the use of pig iron and ferrous scrap derived from both basic oxygen furnaces (BOF) and electric arc furnaces. The production of direct reduction iron (DRI) from iron ore to metallic iron using non-coking coal or natural gas is currently a subject of active research and development. The utilization of DRI is markedly inferior to the consumption of pig iron and scrap. However, a number of factors influence the gradual expansion of both the production and consumption of DRI in steelmaking.

The situation is primarily influenced by the availability of abundant natural gas reserves in conjunction with limited coal reserves, cost-effective electricity generation, and a growing shortage of coking coal and coke. Furthermore, the necessity for natural iron free from impurities to meet rising steel quality demands, limited resources of high-purity ferrous scrap, and a focus on reducing environmental impact further shape the situation.

In addition to the substitution of metallized raw materials for scarce scrap, the intensification of environmental requirements in the global iron and steel industry in recent years has resulted in a surge in demand for premium iron ore raw materials with elevated iron content and minimal impurities. Such products include metallized pellets and hot briquetted iron (HBI). The technology of the direct reduction of iron, which is used in the production of metallized pellets (including DRI and HBI), is regarded as one of the most promising areas for the effective development of global metallurgy.

According to the World Steel Association [1], global production of HBI/DRI during the period from January to May 2024 exceeded 50.81 million tons. Approximately 60% of this production comes from two countries: India, where coal-based technology is widely utilized, and Iran. Russia and Saudi Arabia are the next most prominent producers in terms of production volumes.

A variety of techniques may be employed for the metallization of iron ore materials. The various solid-phase processes for the production of DRI/HBI in different units and utilizing different reducing agents, including gas and solid reducing agents, are outlined in Table 1 [2,3,4].

The American company Midland-Ross developed the direct reduction of iron, a widely used technology, in the mid-1960s. The reduction of iron oxides from pellets or lump ore is conducted in a shaft furnace, where a counter flow of iron-containing material is arranged to descend under its own weight in conjunction with a hot reducing gas. The temperature within the furnace is maintained at a level below that required for the softening of the charge materials [5].

Fe_2_O_3_ + 3H_2_ → 2Fe + 3H_2_O↑, (1)

Fe_2_O_3_ + 3CO → 2Fe + 3CO_2_↑. (2)

The reducing gas is hydrogen, which is produced in conjunction with carbon monoxide as a result of the conversion of natural gas in a separate reactor, referred to as a reformer.

CH_4_ + 2O_2_ → CO_2_ + 2H_2_O. (3)

Each metallization plant is comprised of a shaft furnace, a natural gas reformer, an inert gas production system, an aspiration system, and auxiliary systems. The shaft furnace is comprised of a feed hopper, an upper gate with a charge distributor, a lower gate, and a pendulum feeder for the discharge of the metallized product. The furnace is divided into three distinct zones, namely a reducing zone, an intermediate zone, and a cooling zone, each of which is characterized by a specific height. 

The metallized pellets are cooled to a temperature of between 40 and 50 degrees Celsius in the lower part of the furnace and subsequently discharged by feeders. Following this, the pellets are screened in order to remove any fines. The cooling process is conducted using a gas mixture comprising reducing gases and fume-mine gases. Subsequently, the metallized product is employed in the production of steel, typically within electric arc furnaces. The utilization of waste gas heat is achieved through the use of recuperators, which serve to heat the natural gas and air supplied for fuel combustion in the reformer [5].

The depth of the reduction process can be gauged by the degree of metallization, which is determined by the Equation (4):

φ_met_ = Fe_met_/Fe_total_ · 100%. (4)

In this context, the term φ_met_ denotes the degree of metallization, expressed as a percentage. The variables Fe_met_ and Fe_total_ represent the contents of metallic iron and total iron, respectively, in the metallized product.

The MIDREX process is widely regarded as the most reliable and productive direct reduction iron technology in the world. Roughly 80% of global direct reduced iron production is attributed to the MIDREX process and its analogous variants, with natural gas employed as the reducing agent. The remaining 20% is produced in facilities that manufacture sponge iron, a derivative of DRI, using coal.

The resulting metallized pellets, comprising between 90 and 96.97% iron, can be utilized directly in the electric steelmaking process. The storage and transportation of DRI in its original form presents significant challenges, primarily due to its tendency to oxidize rapidly [6]. Consequently, direct iron reduction plants typically incorporate an additional production step, namely hot briquetting. Briquettes of 60–200 cm^3^ are not subject to such rapid corrosion and can be stored in open areas and transported over long distances.

The utilization of pellets in the processes of direct iron production [7,8] is subject to rigorous specifications. These specifications include the recommendation and availability of pellets with a FeO content of less than 1.0% and a total iron content exceeding 65%. Additionally, the recommended and available content of the 9–15 mm fraction is set at a minimum of 85%, with a maximum of 50% for the 9–11 mm fraction. Furthermore, the required strength of the pellets must exceed 300 kg/pellet, which is above the standard compressive strength for blast furnace pellets (200–250 kg/pellet) [9,10]. Moreover, the utilization of ore-coal pellets is a recognized phenomenon [11,12].

The following known results and research works on the processes of direct iron production from titanomagnetite raw materials have been identified. The direct reduction of titanomagnetite concentrate was achieved through the utilization of Itmk3 technology, in conjunction with additives at a concentration of 8–10%. The production of metallized pellets with an iron content of 92.9% was achieved through the use of CaF_2_ and low-grade coal with an ash content of 35% at a quantity of 15–16%. These pellets are suitable for replacing scrap in electric furnaces [13].

The results presented in Ref. [14] demonstrate that under conditions of a reducing atmosphere and complete carburization, effective separation of the slag and metal phases is achievable. This results in the transfer of over 95% of Fe into the metal phase, while over 90% of Ti remains in the slag.

An increase in process temperature favors the reduction of vanadium oxides, leading to an increase in the mass fraction and degree of V reduction [15].

The Fe–Ti separation efficiency first increases and then decreases with increasing P(H_2_)/P(H_2_ + CO) [16,17].

An increase in the MgO content has been demonstrated to enhance the vanadium reduction ratio, while achieving a low titanium content in molten iron is contingent upon a MgO content in slag that is below 11% [18].

As the basicity of CaO/SiO_2_ increases during the smelting of metallized titanium-magnetite pellets, the reduction factors of Fe, V, and Cr increase, and the transition of TiO_2_ into slag first increases and then decreases [19,20].

Two pathways are available for the reduction of iron- and titanium-bearing oxides to metallic iron: the reduction of titanohematite to titanomagnetite, followed by wüstite and finally metallic iron [21]. Similarly, the reduction of pseudobrookite to metallic iron occurs.

The analysis of the mechanisms involved in these processes indicates that the mineral composition and microstructure of the titanomagnetite pellets have a significant influence on their reducing behavior. Furthermore, the properties of the annealed pellets can be enhanced by the addition of ordinary magnetite [22].

It is possible to utilize waste materials containing, for example, cobalt (Co) as an additive to vanadium-containing pellets. The addition of Co_2_O_3_ promotes aggregation and the diffusion of metallic iron particles [23].

It is also important to note the role of the reducing gas in these processes (CO/H_2_ ratio), which allows for achieving a metallization degree above 90% for both metallized magnetite and titanomagnetite pellets [24,25].

Titanomagnetite ores represent a valuable source of not only iron, but also titanium and vanadium, which are in increasing demand within the industrial sector. The complex nature of this ore also makes its extraction more cost-effective and optimizes the recovery of iron and titanium.

The objective of this research is to investigate the potential of utilizing titanomagnetite concentrate as a raw material for the production of iron ore pellets and their subsequent metallization, with the aim of achieving the requisite characteristics for use in both blast furnace and electric steelmaking processes.

## 2. Materials and Methods

In this study, we examined a low-grade iron ore titanomagnetite concentrate derived from ore enrichment at the deposit, Pervouralsk, Sverdlovsk region, Russian Federation. The ores at the deposit can be classified into two principal categories: massive (solid, 2.2%) and disseminated (97.8%). The most prevalent ore type is the poor disseminated ore, which constitutes the majority of the ores present. The principal ore minerals are titanomagnetite and ilmenite, with minor concentrations of hematite, chalcopyrite, pyrite, bornite, and chalcocite. Non-ferrous minerals are mainly represented by hornblende, pyroxene, plagioclase, and chlorite, with smaller amounts of epidote, garnet, sphene, and spinel. In some cases, rare grains of apatite and veins of calcite are also present. Titanomagnetite is the primary ore mineral.

The initial ore was crushed according to a three-stage scheme to a size of −70 + 0 mm. Subsequently, the crushed ore was subjected to screening on a 10 mm sieve, whereby the products falling within the ranges of −70 + 10 mm and −10 + 0 mm were separated. Both products were then subjected to distinct dry magnetic separation processes, resulting in the production of magnetic products (poor concentrates) and non-magnetic products. Following the upgrading process, which included fine grinding and a two-stage, low-intensity magnetic cleaning procedure, a vanadium titanomagnetite concentrate was produced [26]. The magnetic products were combined to form a total poor iron concentrate with a total iron content of at least 32%.

Two enrichment schemes were investigated and evaluated in laboratory settings to produce iron ore titanomagnetite concentrates, designated as No. 1 (65% Fe_total_) and No. 2 (68.5% Fe_total_), both of requisite quality.

To pelletize the iron ore titanomagnetite concentrates, a laboratory drum-type pelletizer was utilized, allowing for adjustments to both the angle of inclination and the speed of rotation. The technical specifications of the pelletizer are outlined in Table 2.

The binder used for the pelletization of both batches of pellets was bentonite clay, with an additive concentration of 0.7% by mass. Additionally, up to 5–7% of pure water was employed.

Subsequently, the pellets were dried in a drying cabinet at a temperature of 110 °C for a period of two hours. Following this, the pellets underwent high-temperature firing under the following conditions: heating to 1300 °C in air for 120 min, exposure for 15 min, and cooling with the furnace. The height of the pellet layer in the substrate was maintained at 25 mm throughout the process. The firing of the dry pellets was conducted in a muffle furnace, which was equipped to adjust and maintain the required heating rate.

**Figure 1:**
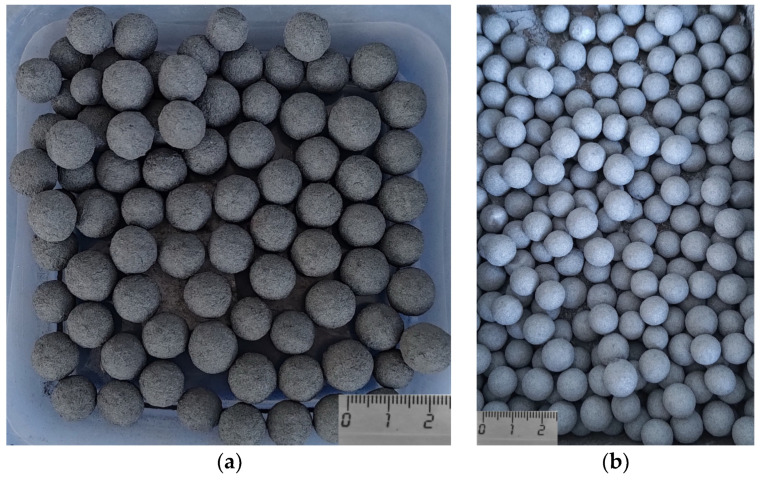
A photograph of the annealed pellets: (**a**) total iron content: 64.5%; (**b**) total iron content: 68.5%.

The images displayed in Figure 1 illustrate the pellets of standard quality (a) and improved quality (b), respectively.

The chemical analysis was conducted using a titrimetric method. The determination of Fe_total_ was performed in accordance with the standards set forth in the Russian National Standard 32517.1-2013, while the determination of Fe_met_ and FeO was conducted in accordance with the standards set forth in the Russian National Standard R 53657-2009.

The phase composition of the samples was determined using an X-ray diffraction (XRD) instrument, the XRD-7000S (Shimadzu, Kyoto, Japan), with CuKα radiation and a graphite monochromator. The identification of phases was conducted using the PDF4 database, which is maintained by the International Centre for Diffraction Data (ICDD) in Pennsylvania, USA.

To study the mineralogical composition, the samples were subjected to micro-X-ray structure analysis using a scanning electron microscope (SEM) and an optical microscope. The SEM was a model JSM-5900LM, from Jeol Ltd., Akishima, Tokyo, Japan, while the optical microscope was an Olympus GX-51 from Shinjuku, Kyoto, Japan.

The analysis of concentrate particle size (including the smallest diameter, length, and average diameter) and shape was conducted using a Camsizer XT (Retsch Technology GmbH, Haan, Germany) equipped with the X-Change modular system. The standard measurement range was from 1 µm to 3 mm. The imaging duration was 40 s, with an imaging speed of 1:1 and a dispersion pressure of 50 kPa.

Furthermore, samples of the initial concentrate and those resulting from the beneficiation process were dispatched for analysis to ascertain the elemental composition of both the concentrate and its individual grains. Investigations were conducted using an scanning electron microscope, the VEGA LMS (fourth generation with a thermoemission, field-emission cathode), manufactured by TESCAN in the Czech Republic. This was coupled with an energy dispersive analysis attachment, the Xplore30, produced by OXFORD Instruments. This setup enabled the acquisition of SEM images and real-time analysis of the elemental composition.

To comply with the requirements set out in ISO 4700, compression tests were conducted on a universal machine (BT1-FR050THW.A1K, Zwick GmbH, Ulm, Germany) with a displacement speed of 10 mm/min, and the resulting deformation diagram was duly recorded. Prior to the compression test, the pellets were individually weighed on an electronic scale (ATL-220d4-1, ACCULAB, Nouvelle-France, Mauritius) with an accuracy of ±0.0003 g to determine their mass.

The study of the reducibility of pellets No. 1 (for blast furnace production) was conducted on the unit in accordance with the requirements set forth in the Russian National Standard 17212-84 (ISO 4695 analogue). The method entails the reduction of the sample by carbon monoxide under specified temperature conditions (heating up to 1100 °C), followed by the determination of the degree of reduction based on the results of chemical analysis of the initial and reduced samples or the loss of oxygen mass during reduction. The composition of the reducing gas is CO (33 ± 0.5%) and N_2_ (65 ± 0.5%), with a flow rate of reducing gas into the reaction chamber of (30 ± 1) dm^3^/min. The heating mode employed for the reduction of the sample is as follows: the sample is heated for the first 40 min at a rate of 15 degrees Celsius per minute up to 600 degrees Celsius, and then for the subsequent 175 min at a rate of 2.86 degrees Celsius per minute up to 1100 degrees Celsius.

The study of strength at low temperature disintegration (LTD) was conducted in accordance with the international standard ISO 13930. The essence of the method is the reducing of the sample by a gaseous reducing agent in a rotating drum at a specified temperature regime, followed by sieving of the test material into size classes (+6.3 mm; +3.15 mm; +0.5 mm; −0.5 mm), which allows for the characterization of its strength properties. The composition of the reducing gas is as follows: CO (20 ± 0.5%), CO_2_ (20 ± 0.5%), H_2_ (2%), and N_2_ (58 ± 0.5%). The temperature of the process is 500 degrees Celsius.

The investigation of the temperature range associated with the softening and melting of pellets was conducted. This technique entails subjecting a sample of the test material to heating in an inert gas environment, with the objective of determining the temperature at which softening commences, as indicated by the immersion of a rigid rod into the sample under the influence of external pressure. Additionally, the technique allows for the delineation of the temperature range encompassing the onset of softening.

The conditions of the metallization process were as follows. The composition of the reducing gas was CO/N_2_ at a ratio of 90/10%. The heating and holding temperature was set at 1050 °C, with a heating rate of 10 °C/min and a holding time of 3 to 4 h. The mass of the pellets was 100 g, and the pellets were cooled in a nitrogen medium until reaching 150 °C.

The pellets were loaded into a mesh basket, which was installed in an electric tube furnace. The flow of reducing gas was supplied to the furnace from the bottom to the top. The mass of the pellets was determined both before and after the metallization process. Subsequently, the cooled pellets were prepared for chemical, phase, and mineralogical analyses.

## 3. Results and Discussion

The chemical composition of the initial concentrate, as well as that of concentrates No. 1 and No. 2, is provided in Table 3.

**Figure 2:**
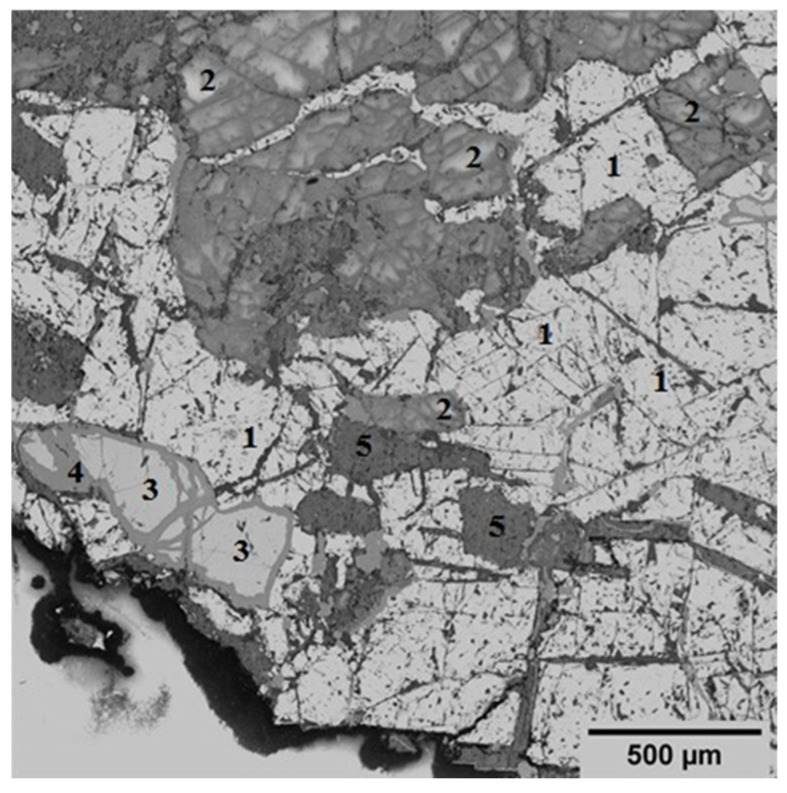
Specific electron microscopic image that showcases the structural details of an initial concentrate using back-scattered electrons (BSE). Symbols: 1—magnetite; 2—plagioclase; 3—ilmenite; 4—sphene; 5—tremolite.

The mineralogical composition of the initial concentrate is diverse, with a predominant presence of magnetite (Fe_3_O_4_) accounting for 51.4%. This phase is identified as a light crystalline structure (Figure 2), indicating its significance in the concentrate’s overall mineralogy. In addition to magnetite, the concentrate contains 32.58% layered aluminosilicate structures, specifically chlorite-serpentine, which is represented by the formula (Mg,Al)(Si,Al)_4_O_10_(OH)_8_. This component highlights the presence of secondary minerals that can influence the concentrate’s properties and processing behavior. Plagioclase, a common feldspar mineral with the formula (Ca,Na)(Si,Al)_4_O_8_, is also found within this group. Minor phases include ilmenite (FeTiO_3_), which is present at up to 7%, and sphene (CaSiTiO_5_) at up to 2%. These minerals are typically associated with titanium and can play a role in the concentrate’s potential applications and value. Additionally, tremolite, characterized by the formula Ca_2_Mg_5_Si_8_O_20_(OH)_2_, is observed in minor inclusions, comprising up to 6% of the concentrate. This amphibole mineral may affect the concentrate’s physical and chemical properties, particularly in terms of its thermal stability and reactivity.

**Figure 3:**
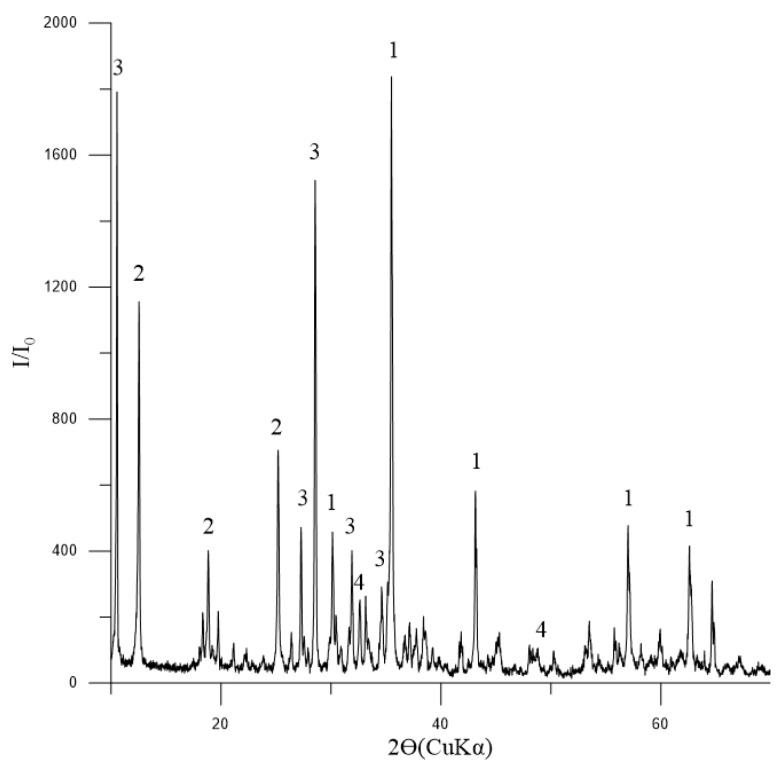
Diffractogram of the initial concentrate: 1—Fe_3_O_4_ (magnetite) [01-088-0315]; 2—(Mg,Al)_6_(Si,Al)_4_O_10_(OH)_8_ (chlorite-serpentine) [00-052-1044]; 3—Ca_2_Mg_5_Si_8_O_22_(OH)_2_ (tremolite) [00-013-0437]; 4—FeTiO_3_ (ilmenite) [01-075-0519].

The phase composition of the initial concentrate, as depicted in Figure 3, indicates that magnetite is the dominant mineral present. Additionally, the presence of tremolite and chlorite-serpentine indicates that there are also silicate minerals involved. The minor quantity of ilmenite suggests that titanium is also present in the concentrate.

**Figure 4:**
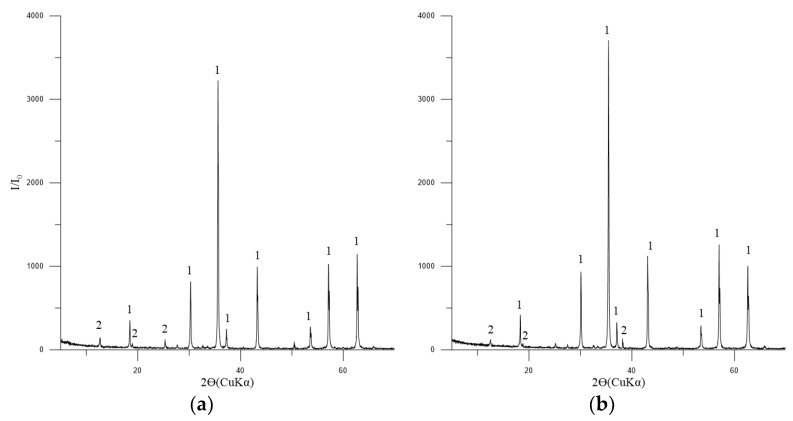
Diffractogram of the concentrates No. 1 (**a**) and 2 (**b**). Symbols: 1—Fe_3_O_4_ (magnetite) [01-088-0315]; 2—(Mg,Al)_6_(Si,Al)_4_O_10_(OH)_8_ (chlorite-serpentine) [00-052-1044]; FeTiO_3_ (ilmenite) [01-075-0519].

The principal phase in the concentrates is magnetite, with tremolite, chlorite-serpentine, and trace amounts of ilmenite also present (Figure 4).

**Figure 5:**
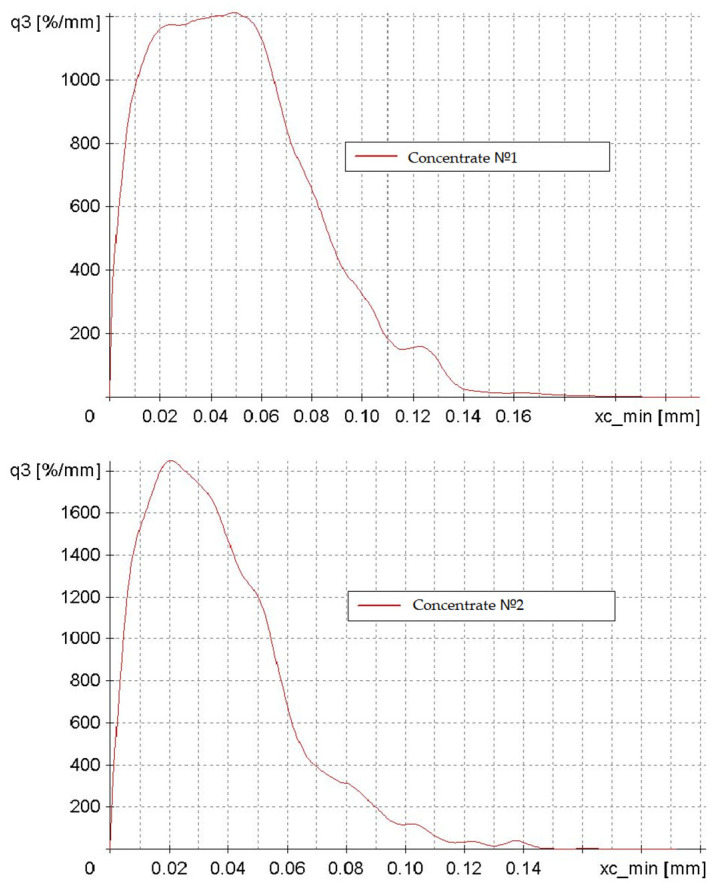
Particle distribution density plots of concentrates No. 1 and No. 2, categorized by particle size from 0 to 0.15 mm.

The investigation into the particle size and shape of concentrates No. 1 and No. 2 yielded the following findings (Figure 5). The abscissa axis (xc_min) represents particle size in millimeters, while the ordinate axis (q3) depicts particle distribution density as a percentage per millimeter.

The two graphs illustrate a modal distribution with negative asymmetry, indicating that the predominant particle size of the concentrate is 0.02–0.05 mm. The particle size distribution of Concentrate No. 1 is more heterogeneous, featuring a broader range of particle sizes and a less distinct peak. The maximum density of the distribution is characteristic of Concentrate No. 2. This discrepancy can be attributed to the different enrichment methodologies employed in the initial preparation of the low-grade concentrate.

**Figure 6:**
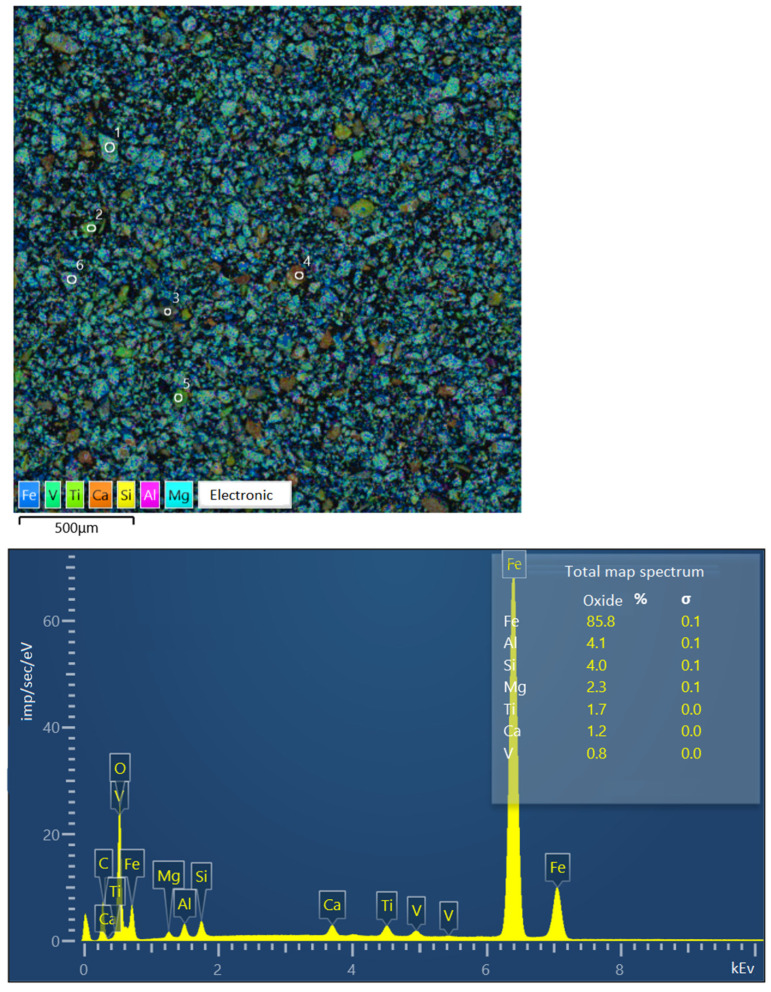
Image of concentrate sample No. 2 and elemental spectra.

Figure 6 presents the findings from the image of the sample of Concentrate No. 2 (the studied large particle grains are marked with numbers) and the spectra of individual elements converted to oxides. Iron is converted to FeO, as specified in the software SEMVIEW 8000, while the remaining elements are converted to standard oxides.

The study focused on examining a series of large grains, measuring approximately 0.071 mm in size. The grains were classified into four categories (see image on the Figure 6, symbols from 1 to 6):

1. A titanomagnetite particle with ilmenite ingrowth.
2. A particle of presumably pure ilmenite.
3. A poorly formed particle, exhibiting a sprout structure and comprising 19.36% Fe.
4. A rock particle, comprising 5.64% Fe.
5. A particle of presumably pure ilmenite.
6. A particle of presumably pure titanomagnetite.

Table 4 presents the results of the elemental composition determination of the concentrate sample of improved quality and the individual large grains contained therein. Additionally, the table presents the findings of the chemical analysis of titanomagnetite grains, representing the physically separated monomineral fraction of titanomagnetite.

The iron content of the concentrate, as determined by electron microscopy, was found to be 68.73%, which is in close agreement with the results of the chemical analysis (68.5% Fe).

The chemical composition of the pellets obtained is presented in Table 5.

**Figure 7:**
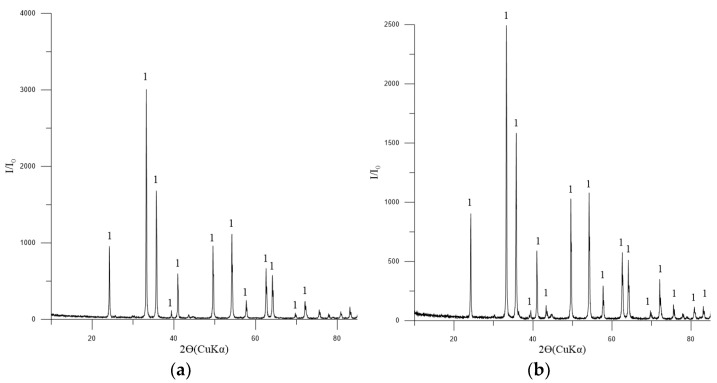
Diffractogram: (**a**) pellets No. 1; (**b**) pellets No. 2. Symbols: 1—Fe_2_O_3_ (hematite) [01-071-5088], minimum of Mg_2_SiO_4_ [01-075-1450] and CaSiO_3_ [01-075-4984].

The analysis of the pellets reveals that hematite is the principal mineral present, with minor components including Mg_2_SiO_4_ and CaSiO_3_ (Figure 7). 

**Figure 8:**
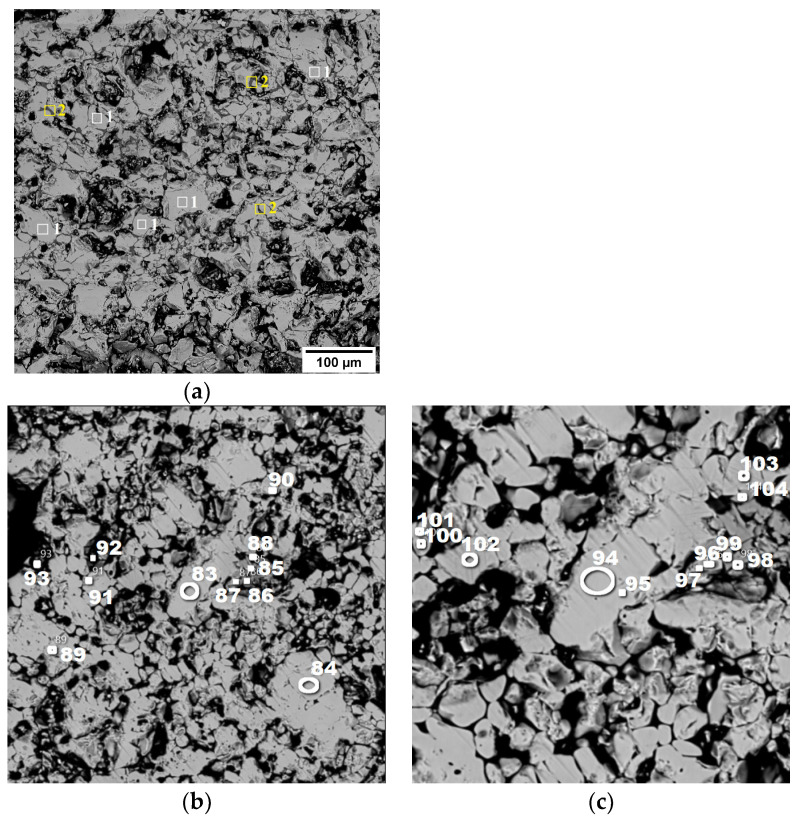
Structure of pellets No. 1 (**a**) and No. 2 (**b**,**c**) (in back-reflected electrons) at magnifications of 100× (**a**), 50× (**b**,**c**).

In pellet No. 1, hematite (1) is the dominant phase, accompanied by a small amount of complex silicoferrite (2), specifically Ca_4.2_Mg_4_Fe_10.3_Si_9.5_O_40_. This is visually represented in Figure 8a.

For pellet No. 2, the mineralogical composition is detailed in Figure 8b,c. This pellet also prominently features hematite, as indicated by the spectra labeled (83, 84, 89–95), and (101–103). Additionally, there is a minor presence of Fe_2_TiO_5_, known as pseudobrookite, which can be identified in spectra (85–88, 96–99), and (104).

The findings suggest that the firing conditions applied during the processing of these pellets led to the complete oxidation of magnetite to hematite, resulting in the observed mineralogical compositions. 

One of the crucial metallurgical attributes of pellets is their compressive strength. The compressive strength of annealed pellets exhibits considerable variation between different manufacturers, with reported values ranging from 150 to 300 kg/pellet [27,28].

Given the significant alteration in the contact area between the deformed pellet and the testing apparatus during compression testing, it is not appropriate to utilize destructive normal contact stress as a representative characteristic of strength, as is the case in compression tests of cylindrical specimens. Furthermore, the results of mathematical modeling [29,30] indicate that the most unfavorable stress state in the compression of spherical-shaped pellets occurs in the center due to the action of intense tensile radial stresses.

Consequently, the magnitude of the breaking force in compression tests is typically employed as a metric for assessing the strength of pellets. Nevertheless, a number of authors (e.g., [31]) posit that the compressive fracture energy is a more representative indicator of pellet strength. This is because the energy criteria of strength, in contrast to the force criteria, permit consideration of the dissipation of energy during the plastic deformation of the material.

In its initial unburnt state, the pellet is composed of iron ore magnetite concentrate and a binder, namely bentonite. Upon firing, the magnetite is oxidized, resulting in the formation of hematite, which represents the active phase. As a consequence of the sintering of the hematite grains, the strength characteristics are enhanced [32]. If the process of magnetite oxidation has occurred completely over the entire cross-section of the pellets, the resulting structure will be homogeneous and composed of hematite.

In industrial conditions, pellets are roasted at temperatures of 1250–1350 °C, with a typical heating rate of 100 °C/min or higher. At such firing parameters, complete oxidation of magnetite does not occur, and pellets typically exhibit a zonal structure [33,34,35,36] comprising a magnetite core and a hematite shell, thereby displaying a pronounced two-phase structure. The disparity in temperature between the magnetite and hematite zones of zonal pellets, which undergo a transition from a plastic to an elastic state, is accompanied by a notable contraction and a variety of alterations in their dimensions. This phenomenon gives rise to the emergence of internal stresses at the interface between the magnetite and hematite zones [37].

**Figure 9:**
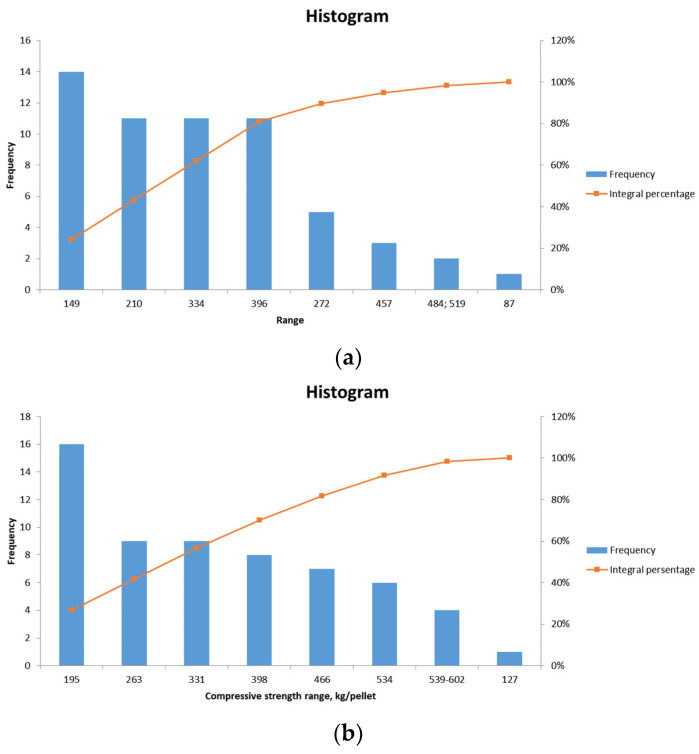
Frequency distribution graph of compressive strength values of pellets No. 1 (**a**), pellets No. 2 (**b**).

The results of the compression tests conducted on pellets No. 1 and No. 2 are presented in Figure 9a and Figure 9b, respectively.

The average strength of laboratory pellets No. 1 (for blast furnace production) is 246.6 kg/pellet, with a minimum of 78 kg/pellet, a maximum of 519 kg/pellet, a median of 240 kg/pellet, and a mode of 320 kg/pellet. The requisite compressive strength of pre-main unfluxed pellets is greater than 220 kg/pellet.

The average strength of laboratory pellets No. 2 (for metallization) is 312.6 kg/pellet, with a minimum of 127 kg/pellet, a maximum of 602 kg/pellet, a median of 295 kg/pellet, and a mode of 252 kg/pellet. The requisite compressive strength of non-fluxed pellets for metallization is greater than 300 kg/pellet.

**Figure 10:**
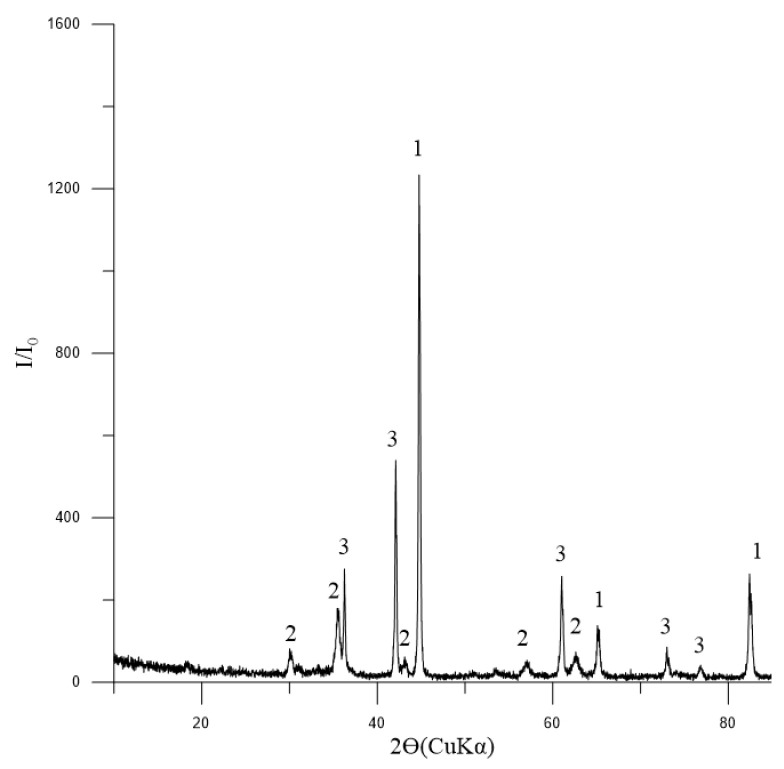
Diffractogram of reduced pellets No. 1: 1—Fe_met_ [01-076-6587]; 2—Fe_3_O_4_ (magnetite) [01-088-0315]; 3—FeO (wüstite) [01-077-7981].

A study was conducted to examine the reducibility of pellets No. 1 (for blast furnace production). The chemical composition of the reduced pellets is as follows: the total iron content was found to be 76.29%, with the iron oxide accounting for 40.46% and the metallic iron component representing 40.34%. The phase composition of reduced pellets No. 1 is presented in Figure 10.

**Figure 11:**
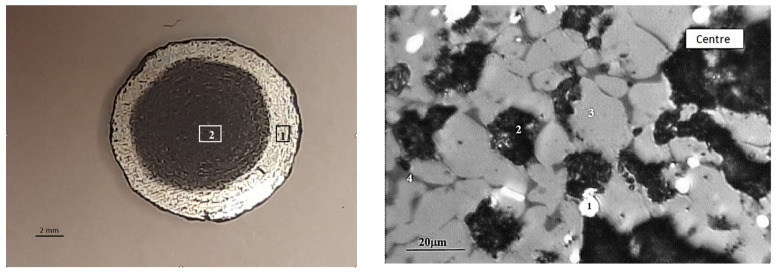
Typical macrostructure of reduced BF pellets: **left** (1—edge, 2—center), **right** (in the center: 1—Fe_met_; 2—pores; 3—magnetite; 4—FeO).

The macrostructure of the reduced pellet is illustrated in Figure 11. It can be observed that the reducing of the pellet occurs in a manner that progresses from the surface to the depth. The absolute degree of reducing by chemical analysis (R1) was 66.56%, while the absolute degree of reducing by mass loss (R2) was 63.38%. The actual degree of reducing (R_fact_) was 66.4%. It is recommended that the reducing rate for pellets should be above 80%. The reducibility of pellet No. 1 is below the recommended threshold, which is to be expected given the low porosity and strength of the pellets. There is an inverse relationship between the strength of iron ore materials and reducibility, whereby an increase in one parameter is accompanied by a decrease in the other [38].

The low-temperature fracture index (LTD_+6.3_) was found to be 37.2%, which is an unsatisfactory result for pellets. It is recommended that the LTD index should be above 60%. The low value of hot strength can be attributed to the insufficient presence of binding oxides in the concentrate, such as CaO, SiO_2_, MgO, and Al_2_O_3_, which are essential for imparting thermomechanical strength [39,40,41,42].

The softening temperature range is 100 °C (1160–1260 °C), representing an average value for the temperature range typically observed in blast furnaces.

Table 6 presents the chemical composition of pellets No. 2 following metallization at 3 and 4 h of exposure. The degree of metallization is calculated as the ratio of metallic iron to total iron, expressed as a percentage. In order to facilitate electrowinning, the metallization degree must exceed 88%.

**Figure 12:**
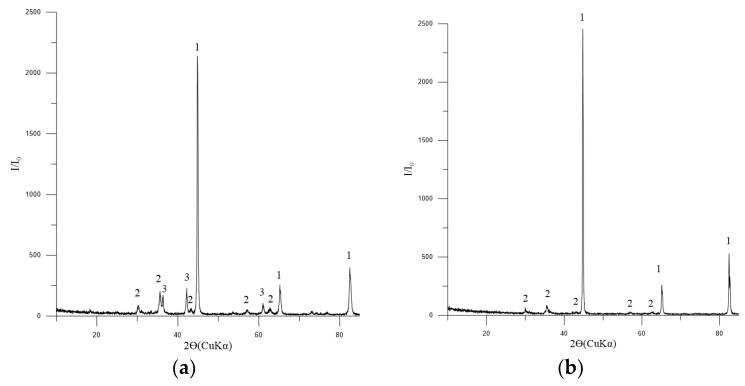
Diffractogram of metallized pellets No. 2: (**a**) 3 h of exposure; (**b**) 4 h of exposure: 1—Fe_met_ [01-076-6587]; 2—Fe_3_O_4_ (magnetite) [01-088-0315]; 3—FeO (wüstite) [01-077-7981].

The phase composition of the metallized pellets is shown in Figure 12a,b.

**Figure 13:**
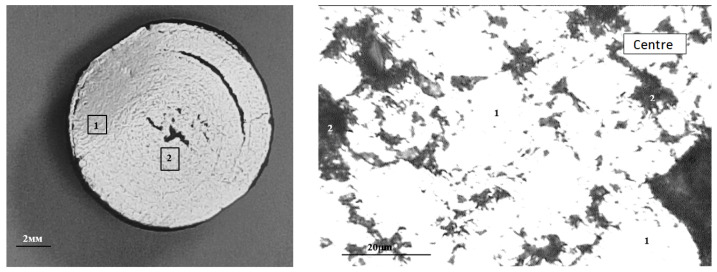
Typical macrostructure of metallized pellets: **left** (1—edge, 2—center), **right** (in the center: 1—Fe_met_; 2—pores).

The macrostructure of the metallized pellets is shown in Figure 13.

## 4. Conclusions

The following results were obtained from the study.

The chemical, phase, and mineralogical compositions of concentrates of standard (64.5% Fe) and upgraded (68.5% Fe) quality from the Pervouralskoye deposit were analyzed. The principal phase identified in the concentrates is magnetite, with tremolite, chlorite-serpentine, and a minor quantity of ilmenite also present.

Under laboratory conditions, pellets of standard and superior quality were produced and subjected to combustion. The principal phase present in the pellets was hematite.

The compressive strength of both types of annealed pellets was evaluated. For pellets intended for blast furnace production (standard, No. 1), the average compressive strength was 247 kg/pellet, which complies with the requirements for blast furnace production. For pellets designed for metallization (superior quality, No. 2), the average compressive strength was 313 kg/pellet, which complies with the requirements for the metallization process.

The metallurgical properties of pellet No. 1 were investigated. The actual reducing degree (R_fact_) was found to be 66.4%, which is below the recommended value for blast furnace production. Similarly, the low-temperature disintegration index LTD_+6.3_ was determined to be 37.2%, again falling below the recommended value for blast furnace production. However, the softening temperature range was found to be 100 °C, which meets the requirements for blast furnace production. To impart the requisite metallurgical characteristics to the pellets, it is advisable to test a variety of firing modes and to incorporate a greater proportion of binding materials into the charge prior to pelletization, thereby enhancing the strength of the material.

The degree of metallization of annealed pellets No. 2, following restoration by gas with a composition of CO + N_2_ (90/10%), after 3 h and 4 h exposures, was investigated. The results demonstrate that the optimal degree of metallization was achieved with a four-hour exposure, reaching 92.5%. This level of metallization aligns with the requirements for electric steelmaking production.

## Figures and Tables

**Table 1:** Methods of metallization.

Type of Reducing Agent	Unit
Reducing gas	Shaft furnaces Midrex, HYL-III, HYL-IV M, Armco, Purofer, Arex, Ghaem, Plasmared, NSC Retorts HYL-I, HYL-II, Hoganas Fluidised bed reactors (FBRs) FINMET, FIOR, Spirex, Circored Rotary kilns FASTMET, ITmk3
Solid reducing agent	Shaft furnaces BL, KINGLOR, METOR, NML Rotary kilns SL/RN, CODIR, ACCAR, DRC, TDR, SIIL, OSIL, Jindal Rotary (hearth) kilns INMETCO, FASTMET, Comet

**Table 2:** Technical specifications of the pelletizer.

Parameter	Unit of Measurement	Parameter Value
Diameter	mm	450
Width	mm	250
Angle of inclination	degree	0–45
Rotational speed	rpm	0–60

**Table 3:** Chemical composition of the materials.

Sample	Content, Mass. %											
	Fe_total_	Fe_met_	FeO	CaO	SiO_2_	Al_2_O_3_	MgO	MnO	TiO_2_	V_2_O_5_	S	P
Concentrate No. 1	65.71	-	23.31	1.40	1.94	1.97	0.75	0.17	1.88	0.68	0.010	0.006
Concentrate No. 2	68.79	-	22.85	1.20	1.25	1.30	0.55	0.16	1.35	0.70	0.0079	0.008
Initial concentrate	38.79	-	12.21	6.64	18.34	10.46	6.77	0.24	4.48	0.44	0.093	0.025

**Table 4:** Elemental composition of concentrate sample and individual grains according to electron microscopy data.

Content, at. %												
Fe_total_	FeO	Fe_2_O_3_	MgO	Al_2_O_3_	SiO_2_	CaO	TiO_2_	V_2_O_5_	MnO	Ni	Co	Losses During Calcination
Total sample of enhanced concentrate												
66.73	–	–	2.33	4.14	4.03	1.16	1.68	0.82	–	–	–	–
4—rock particle (5.64% Fe)												
5.64	–	–	0.37	30.19	40.87	21.14	–	0.17	–	–	–	–
3—poor particle: titanomagnetite aggregate with rock minerals (19.36% Fe)												
19.36	–	–	22.58	21.39	29.64	0.67	0.69	–	0.12	–	–	–
2 and 5—particles of presumably pure ilmenite												
36.97	47.56	–	–	0.61	0.46	–	49.08	–	2.28	–	–	–
38.39	49.39	–	–	0.61	0.26	–	46.45	0.72	2.57	–	–	–
1—titanomagnetite particle with ilmenite ingrowth												
67.01	–	–	1.91	5.09	0.33	–	5.03	1.09	0.33	–	–	–
6—particle of presumably pure titanomagnetite												
68.88	–	–	–	7.58	0.36	–	2.00	1.24	0.20	–	–	–
Monomineral fraction of titanomagnetite												
68.32	31	63.5	0.35	1.3	0.1	0.1	2.9	0.88	0.16	0.01	0.012	0.3

**Table 5:** Chemical composition of laboratory pellets No. 1 and No. 2.

Sample	Content, Mass.%										
	Fe_total_	Fe_met_	FeO	CaO	SiO_2_	Al_2_O_3_	MgO	MnO	TiO_2_	V_2_O_5_	S
Pellets No. 1	64.58	-	1.20	1.328	1.76	1.63	0.91	0.087	1.47	0.70	0.006
Pellets No. 2	67.49	-	<1.00	1.200	0.73	1.22	0.66	0.080	1.24	0.70	0.003

**Table 6:** Chemical composition of metalized pellets.

Time of Exposure	Content, Mass. %			Degree of Metallization, %
	Fe_total_	FeO	Fe_met_	
3 h	83.53	7.34	72.02	86.22
4 h	85.00	0.10	78.62	92.50

## Data Availability

The original contributions presented in this study are included in the article. Further inquiries can be directed to the corresponding author.
